# Can immunological principles and cross-disciplinary science illuminate the path to vaccines for HIV and other global health challenges?

**DOI:** 10.1098/rstb.2014.0152

**Published:** 2015-06-19

**Authors:** Christopher B. Wilson, Christopher L. Karp

**Affiliations:** Global Health Program, Bill & Melinda Gates Foundation, 500 Fifth Avenue North, Seattle, WA 98109, USA

**Keywords:** vaccine, HIV, malaria, antibody, avidity, immune

## Abstract

Vaccines are one of the most impactful and cost-effective public health measures of the twentieth century. However, there remain great unmet needs to develop vaccines for globally burdensome infectious diseases and to allow more timely responses to emerging infectious disease threats. Recent advances in the understanding of immunological principles operative not just in model systems but in humans in concert with the development and application of powerful new tools for profiling human immune responses, in our understanding of pathogen variation and evolution, and in the elucidation of the structural aspects of antibody–pathogen interactions, have illuminated pathways by which these unmet needs might be addressed. Using these advances as foundation, we herein present a conceptual framework by which the discovery, development and iterative improvement of effective vaccines for HIV, malaria and other globally important infectious diseases might be accelerated.

## 1. Introduction

Along with improved sanitation and antibiotics, the development and deployment of vaccines have led to a dramatic decline in death and disability due to infectious diseases over the past century. These benefits have not been equally shared across the globe. Although economic improvement and social equity efforts—such as the provision of vaccines through the GAVI alliance—have helped to more broadly distribute the benefits of vaccines to those in resource-limited settings where the need is greatest [1,2], much remains to be done.

The timeline from identification of the causal agent to the registration and deployment of an effective vaccine has historically been quite long, with a few notable exceptions—the inactivated whole-cell pertussis vaccine (1914) and live-attenuated measles vaccine (1963) were launched within 8–10 years of the discovery of the causative agents. In the case of measles, this best-case scenario was the apparent result of an unusual confluence of events, including the development of methods to isolate and propagate viruses in tissue culture and competition between Salk and Sabin in the race to produce a polio vaccine. The development by Sabin of the live-attenuated polio vaccine illuminated the pathway by which the live-attenuated measles vaccine was developed.

By contrast, we remain without a vaccine for malaria, now more than a century after the causative agent was identified, and as yet have no vaccine for HIV more than 30 years after its discovery. The bar to producing an effective vaccine for these diseases is high. To protect individuals and also prevent them from transmitting the infection to others, vaccines for HIV and malaria must do something that the infection itself does not. By contrast, most vaccines in current use act by mimicking natural infection, although there are precedents for vaccines that protect more effectively than does the natural infection—tetanus, diphtheria, human papilloma virus and bacterial polysaccharide-conjugate vaccines do so. For the latter, it is sobering to note that the fundamental immunological principle that underlies their dramatically greater immunogenicity and protective efficacy compared with natural infection was reported in 1931 [3,4]. However, the first polysaccharide-conjugate vaccine was licensed for use in the United States in 1988, and these vaccines were not deployed to any substantial degree in low-income settings until the launch of the GAVI alliance in 2000. Surely the interval from fundamental insight to application for human benefit can and must be accelerated.

Herein are described approaches by which the discovery, development and iterative improvement of vaccines for globally important infectious diseases might be accelerated. These approaches are based on an emerging understanding of immunological principles operative not just in model systems but in humans, in concert with the development and application of powerful new tools for profiling human immune responses. We further take it as axiomatic that progress will be more efficiently achieved when scientific siloes are breached to promote cross-disciplinary action and to create consortia of collaborators with the commitment and collective expertise needed to advance the discovery and development of vaccines for these unmet needs. Sections 2–4 use HIV vaccines that aim to induce broadly neutralizing antibodies (bNAbs) as an exemplar, and §5 discusses how such principles might also inform the development of a highly effective malaria vaccine and more durably effective pertussis vaccines.

## 2. Broadly neutralizing antibodies to HIV

HIV is a formidable yet not insurmountable foe. HIV can rapidly establish a latent reservoir within CD4-expressing cells of the immune system. This reservoir appears not to be recognized and targeted by the immune system and to be unassailable through anti-retroviral therapy alone. Nonetheless, recent data suggest that antibodies that broadly neutralize HIV viruses can, in primate model systems, prevent the establishment of infection following vaginal or rectal challenge [5–7]. Such antibodies are produced in response to HIV infection, but only in a minority (less than 20% and approx. 1–2% produce antibodies with moderate or exceptional breadth and potency, respectively) of infected individuals [7,8]. Further, when bNAbs do develop, it is many months to years after infection has been established—by then too late to clear the diverse swarm of viral variants that have arisen in these individuals.

Until recently, the technologies, clinical material and cross-disciplinary scientific consortia needed to define the nature and ontogeny of bNAbs were not available. However, prescient clinical scientists with the support of willing patients, funding agencies and others have collected cohorts of individuals followed from before infection was acquired. Within these cohorts, sequential samples from individuals who went on to develop bNAbs provided the essential substrate for discovery. In turn, high-throughput sequencing of viral isolates and immunoglobulin heavy and light chain genes from single B cells or B cell clones from these individuals have revealed the starting points, tempo and evolutionary pathways by which bNAbs developed.

This information is necessary but not sufficient to illuminate the mechanisms by which bNAbs are naturally induced. Advances in structural biology—including X-ray crystallography and high-resolution cryo-electron microscopy—were also essential, as were the computational capacity and tools through which sequencing and structural information could be synthesized into a whole that is vastly greater than the sum of the individual parts. The fruits of this work—and the rate at which new information has arisen—demonstrate the value, importance and co-dependencies of this multi-faceted approach to the problem. Challenges yet remain to turn this knowledge into a practical strategy by which to induce bNAbs that is less lengthy and arduous than occurs in natural infection. Mathematical modelling, modelling informed design of experiments in model systems [9] and exploratory human research trials may help to accelerate the rate at which the scientific community can converge on approaches with a higher probability of success.

bNAbs bind to one of at least six regions on the HIV envelope, the most common being glycan-dependent epitopes within the V1–V2 or V3 regions and epitopes within the CD4-binding site; bNAbs that bind to the membrane proximal external region and other sites have been identified at lower frequencies [5,6,10]. bNAbs are characterized by one or more features that are uncommon in antibodies to other pathogens: long (more than 20 amino acids) third immunoglobulin heavy chain complementarity determining regions (CDRH3), high degrees of somatic hypermutation (more than 12 amino acid substitutions in the variable region of the immunoglobulin heavy chain, VH), restricted usage of specific variable domain genes, and auto- or polyreactivity. These unusual features are likely to account in large part for the relative infrequency, prolonged time frame and complex evolutionary pathways by which bNAbs arise in infected individuals.

What drives the selection and evolution of the B and plasma cells that produce HIV bNAbs? Although only two longitudinal evolutionary pathways have been published to date, the shared features seen in these individuals provide strong support for the notion—perhaps not surprisingly—that HIV mutation and B cell lineage selection and evolution are driven in a bilateral, iterative cause and effect relationship [11–13]. Earlier work revealed that one or at most a few viral variants launch infection in an individual [14]. This transmitted/founder virus is targeted by the initial B cell response that occurs in the first weeks to months of infection, which in most individuals leads over time to the generation of antibodies that can neutralize that founder. Such antibodies apply pressure that selects for viruses with envelope mutations that evade these initial neutralizing antibodies. These escape variants in turn drive new rounds of somatic hypermutation in B cells leading to antibodies that neutralize the variants, a process that, when iterated multiple times over months to years can lead to the production of antibodies with improved potency and breadth with which the viral variants in that individual and in other individuals can be neutralized—the defining features of bNAbs.

The two bNAbs lineages whose evolution has been traced (one a lineage of non-CD4 mimicking, CD4-binding site antibodies [13] and the other a V1, V2 glycan-binding antibody [11]) have relatively long CDRH3 regions (20 and 35 amino acids, respectively) and degrees of somatic hypermutation (i.e. 14% and 17% of VH amino acids, respectively) that are greater than nearly all IgG antibodies specific for influenza and other pathogens (average 7–8%) but less than those of many HIV bNAbs, which commonly exceed 30% [7,15,16]. Notably in both of these individuals, the bNAb lineage had little detectable ability to bind and neutralize the initial founder virus. In one individual, it appears that initiation of the bNAb lineage was driven by a viral variant that arose at approximately the seventh week of infection in response to neutralization of the founder virus by another antibody lineage lacking breadth [13]. In the other, it appears that superinfection by another virus, presumably through a subsequent sexual transmission event approximately 15 weeks after this individual's initial infection, triggered evolution of the bNAb lineage [11]. These findings provide an encouraging suggestion that it may be possible to take shortcuts that limit the number of different immunogen variants needed in the vaccine regimen required to induce a bNAb.

## 3. Is it possible to induce broadly neutralizing antibodies by active immunization and in so doing to improve on nature?

It is likely that a complex regimen consisting of more than two to three unique vaccine compositions to be administered sequentially in a prime and boost regimen would not be practical or affordable in resource-limited settings. Thus, because the natural pathway of bNAb induction involves many viral variants emerging over years, the desired HIV vaccine regimen must improve upon and not merely mimic nature. Immunological, virological and structural principles are now emerging to provide some guidance as to how this evolution might be achieved.

Antibody responses to proteins and glycoproteins, such as the HIV envelope glycoprotein, require a productive interaction between follicular B cells and T follicular helper CD4 T cells (Tfh). This interaction takes place in secondary lymphoid tissues and is cognate for both parties [17–19]. Binding of the envelope immunogen to the B cell antigen receptor (surface immunoglobulin) provides one of the signals needed to activate that B cell. Immunoglobulin also serves as the receptor through which that antigen is internalized by the B cell, after which it is processed into peptides that bind to and are presented on class II MHC molecules (HLA-DR, -DQ and -DP in humans) to Tfh. Tfh with antigen receptors specific for these peptide–MHC complexes can bind these complexes, are thereby activated, and then engage in bidirectional signalling with the presenting B cell. Through this engagement, the B cell receives the requisite second signals needed for activation and entry into germinal centres. There, through an iterative process of ‘Darwinian’ selection, B cells whose surface immunoglobulin has the highest affinity for the antigen internalize and present antigens most effectively to Tfh, thereby competing best for T-cell help. This drives their proliferation and somatic hypermutation, and when somatic hypermutation creates a new immunoglobulin with higher affinity for antigen, such cells are preferentially selected to expand in number and to differentiate into memory B cells and antibody secreting plasma cells and may also switch immunoglobulin isotype. This process of affinity maturation helps to ensure that cells with the highest affinity for antigen come to dominate the response over time.

Unlike vaccines in current use, there is an additional challenge for a vaccine aimed at inducing bNAbs—there is no evidence to suggest that induction of high-affinity antibodies for the initial immunogen will give rise to antibodies with high affinity for the diversity of envelopes in nature. One approach to doing so is to create immunogens that incorporate features from multiple variants—engineering mosaics that encompass multiple variants into a single envelope trimer [20]. There is precedent for such an approach—three variants of the factor H-binding protein from meningococcus B were incorporated by structure-guided design into a single molecule that induced protective antibodies to all three variants [21]. Even so, it has not been possible to date to create a mosaic that can select the requisite unmutated immunoglobulin ancestor(s) from which to drive the full evolutionary pathway needed to produce a bNAb lineage. Moreover, in a given individual, broad and potent neutralization arises from antibodies produced by only one or two lineages, the others either being false-starts or helper lineages that have little breadth but do impose restrictions on viral evolution that may foster bNAb development from other lineages [13,22,23]. Thus, unlike vaccines to pathogens with little variance, it appears that a vaccine regimen to induce bNAbs must first generate sufficient diversity within the starting repertoire to provide the substrate from which a lineage with both potency and breadth can be derived in response to subsequent boosting.

**Figure 1.**
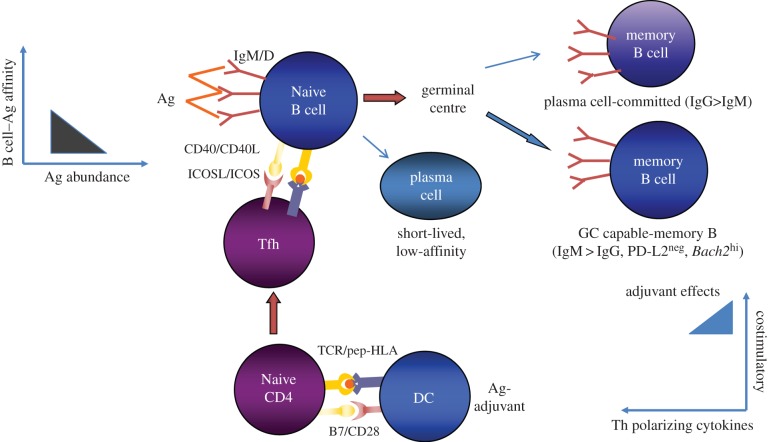
Hypothetical priming immunogen composition for an HIV vaccine designed to induce bNAbs. The primary immunogenic composition is engineered to activate a diverse repertoire of B cells, which preferentially differentiate into memory B cells capable of re-entering germinal centres in response to secondary and tertiary booster immunization rather than inducing short-lived plasma cells and plasma cell-committed memory B cells. Such a composition would contain antigen in moderate abundance and with a multivalent, repetitive structure in order to crosslink B cell antigen receptors (i.e. surface immunoglobulin) with a range of affinities for this primary immunogen. The immunogen structure would be engineered to preferentially engage the unmutated common ancestors of bNAbs rather than narrowly neutralizing antibodies. The adjuvant in the formulation would effectively upregulate MHC class II and costimulatory molecules on antigen-presenting cells and foster the differentiation of Tfh without strongly inducing CD4 T-cell-polarizing cytokines.

**Figure 2.**
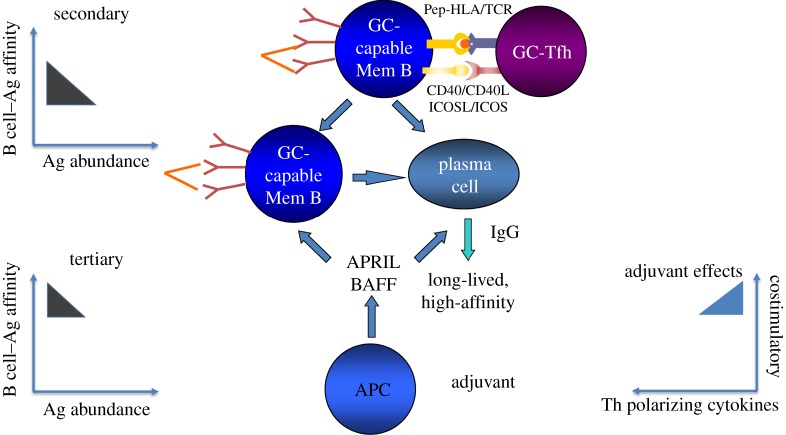
Hypothetical composition for secondary and tertiary immunogenic compositions to foster affinity maturation from a diverse starting repertoire. Progressively limited abundance of antigen and epitope focusing to direct evolution towards higher affinity responses to shared epitopes that can access HIV envelopes at approach angles required to reach their targets in neutralization resistant viral variants. The adjuvant formulation would foster Tfh differentiation [24] and plasma cell longevity [25]. Either the priming or boosting immunogen may need to contain common variants of the target epitope incorporated into the immunogen structure.

How might this be achieved with not more than three different prime–boost vaccine compositions? To achieve breadth at the start, one should in principle provide a sufficient abundance of antigenic epitopes that can engage the unmutated common ancestors of immunoglobulin heavy and light chain pairs that have the potential to give rise to bNAbs. As noted in §2, heavy chains with long CDRH3 loops are more common in bNAbs, in particular in those with more moderate (14–20%) degrees of somatic hypermutation than the extreme degrees (more than 30%) commonly found in bNAbs; these long CDRH3s appear to be present in the unmutated common ancestors giving rise to these bNAbs [11,13,23]. Thus, immunogens that can engage naive B cells with unmutated ancestral immunoglobulin heavy chains with long CDRH3 may be one desirable starting point. The initial vaccine composition would be engineered to contain envelope sequences that can engage multiple such unmutated common ancestors while having somewhat greater affinity for early-stage intermediates on the evolutionary pathway towards known bNAbs within such a lineage than for their unmutated ancestors (figure 1). Boosting immunogens would in turn be engineered to have progressively less affinity for early ancestors and intermediates and progressively greater affinity for the final bNAb, while at the same time progressively constraining the angle of approach to their targets to angles used by the final bNAbs (figure 2). This focusing of the evolutionary pathway along the desired path to the final goal may be achieved in part by modifying glycosylation sites, removing or masking regions to which neutralizing antibodies that lack breadth preferentially bind [22,26] or by other structural design modifications to achieve a similar effect [27].

It will be noted that this approach to improving on nature in order to drive bNAb development [6] aims to recapitulate the pathways by which bNAbs appear to have arisen in the context of natural infection and hence focuses on the engagement of a limited pool of unmutated common ancestors with the potential to give rise to bNAbs. As noted above, there are compelling data suggesting that a major barrier to the development of bNAbs, at least for those reactive with the CD4-binding site and membrane proximal external region, is that the epitopes recognized by these bNAbs are mimics of host epitopes [28–32]. The implications of this hypothesis, to the extent that it is valid, are several. First, it implies that a prime reason for the rarity, lengthy time line for appearance and unusual properties of bNAbs is that B cells with surface immunoglobulin more able to bind directly to these epitopes are efficiently deleted by host tolerance mechanisms during B cell development. Second, it suggests a path by which to drive bNAb development from a much wider pool of B cell precursors that might not need such extensive somatic mutation to yield bNAbs. Extensive literature on epitope spreading suggests that this host tolerance barrier may be surmountable by priming with Env immunogens that are sufficiently similar to HIV-1 neutralizing epitopes to elicit B cell responses with potential to evolve into bNAbs, yet sufficiently dissimilar from the tolerizing epitopes of the host to elicit robust humoral responses. Once primed by such HIV envelope mimetic immunogens, it might be possible to then boost with native Env immunogens—thereby entraining somatic mutation from a much closer structural space.

However, the design of immunogens with a gradient of affinity favouring evolution towards bNAbs is not by itself likely to be sufficient. Rather, it will be necessary to create vaccine compositions that foster the generation of memory B cells that are able to re-enter germinal centres and undergo sequential rounds of affinity maturation in the context of each subsequent booster immunization. Though this clearly occurs in the context of infection and immunization in humans and other species [16], recent evidence in rodents suggests that many if not most memory B cells cannot do so [17,33]. Rather, they are pre-programmed to differentiate directly on subsequent antigen encounter into plasma cells that can rapidly produce the antibody encoded by the precursor memory B cell. Such predisposition is evolutionarily wise in the case of pathogens that have minimal variability in their surface antigens and do not establish latent infection, because on re-encounter a rapid release of antibodies induced in response to the initial infection can prevent disease due to secondary infection. But in the context of an HIV vaccine designed to induce bNAbs, generation of plasma cells from B cells that have not evolved to the point of producing bNAbs would not be useful. Moreover, pre-existing IgG antibodies can block the activation of memory B cells that might be capable of re-entering germinal centres, by epitope masking and engagement of the inhibitory Fc receptor FcγRIIb [17]. Thus, a bNAb-inducing vaccine regimen must induce memory B cells that are able to re-enter germinal centres, re-compete for Tfh help and undergo multiple rounds of somatic hypermutation and affinity maturation [17,19]. Evidence in mice suggests that such B cells may be less differentiated than memory B cells that cannot re-enter germinal centres, i.e. germinal-centre capable memory B cells typically have not switched isotype from IgM to IgG and do not express PD-L2 but do express the transcription factor Bach2 [33,34].

Is enough known about the mechanisms that govern the generation of memory B cells capable of re-entering germinal centres to appropriately engineer vaccine compositions to achieve this goal and to steer the evolution of B cell lineages from unmutated common ancestor to bNAb? Perhaps. Based on current knowledge, primary immunization should promote the generation of substantial numbers of Tfh specific for epitopes within the HIV envelope immunogens—either conserved HIV envelope sequences themselves or heterologous helper epitopes engineered into the immunogens for that purpose. Adjuvants enhance the generation of Tfh by their actions on dendritic cells as well as B cells [35,36]. Abundant Tfh can foster diversity within the initial B cell response so that Tfh are not rationed to the point that only the highest affinity B cells can access help. Similarly, to foster diversity in the starting repertoire, antigen abundance should be moderate rather than limiting and the antigen should ideally be presented in a repetitive or multivalent structure to enable crosslinking of surface immunoglobulin (figure 1). In subsequent booster immunizations, Tfh abundance would be maintained through adjuvant actions; the abundance of antigen would be progressively constrained to drive selection for higher affinity B cells; repetitive structure might also be reduced in all but the ultimate booster, because crosslinking of surface immunoglobulin on memory B cells appears to foster differentiation into plasma cells rather than re-entry into germinal centres [37] (figure 2). Thus, optimal immunogen structure, concentration and composition, and appropriate sequencing of individual immunogens within a prime-boost regimen, are all likely to be required for the induction of bNAbs.

## 4. Can durable antibody responses be induced?

The most successful vaccines in terms of inducing durable responses are live viral vaccines, notably including smallpox and yellow fever vaccines [38]. Recently, systems biological and immunological approaches have shed some light on the molecular mechanisms that account for this durability [39]. However, understanding of the basis of durability remains incomplete—including whether it is pre-programmed during the process of immunization or subject to competitive attrition resulting from subsequent infections, immunizations and other events.

There is also evidence suggesting that the durability of antibody responses to HIV envelope may be particularly short-lived [40]. Moreover, the results of the Rv144 HIV vaccine trial, which is the only human HIV vaccine trial to date that demonstrated protection, indicate that protection waned quickly [41]. This rapid waning is consistent with the observed short half-lives of the overall IgG antibody response and, in particular, of the IgG3 antibody response that appears to be most strongly associated with protection [42].

However, the duration of anti-HIV envelope antibody responses has not been unusually brief in all studies. For example, in the two most recent human trials of a monomeric recombinant HIV gp120 antigen formulated with GlaxoSmithKline's monophosphoryl lipid A-containing AS01 or AS02 adjuvant [43,44], antibody half-life was considerably longer than observed in the Rv144 trial, in which envelope glycoprotein was adjuvanted with alum [42]. Whether the difference in durability reflects differences in the adjuvant or nature or amount of antigen in the composition is not known. However, adjuvants can affect both fate choice between and longevity of memory B and plasma cells and hence the durability of antibody responses, and may also affect antibody isotype, avidity and possibly other qualitative properties of the antibodies. Recent data in mice show that inactivated viral vaccines, live-attenuated microbial vaccines and vaccines that are adjuvanted with alum versus adjuvants containing microbial components use, at least in part, distinct molecular mechanisms to induce and imprint longevity on memory B and plasma cells [25,45,46]. It is likely, though not yet directly demonstrated, that such differences are also true in humans.

## 5. Can the same principles be applied to address other global health challenges?

Though measures to control *Plasmodium falciparum* malaria have resulted in a gratifying decline in numbers of deaths in the recent past, the toll of more than 600 000 per year is still staggering. A vaccine that reduced acquisition and transmission could substantially accelerate progress towards elimination of this disease. Proof-of-principle that a vaccine could prevent acquisition in humans was provided more than 30 years ago, when it was shown that individuals were fully protected from infection following immunization through the bites (approx. 200 bites on five separate occasions) of mosquitoes harbouring *P. falciparum* sporozoites irradiated to prevent replication of parasites in the immunized individuals [47]. This finding has been extended recently by demonstrating that five sequential intravenous inoculations with large numbers of irradiated parasites extracted from infected mosquitoes was similarly effective [48] and that individuals who were immunized through bites from a small number of mosquitoes harbouring virulent parasites and treated to clear the infection before patent parasitaemia developed were also protected from subsequent infection [49]. While encouraging, these whole sporozoite approaches are not likely to be practical and affordable at scale in settings where the burden of disease is greatest. It should also be acknowledged that despite decades of the study of whole sporozoite approaches to vaccination, it remains to be convincingly demonstrated that broad, cross-strain protection is achieved—in this infection in which antigenic variability is a notable hurdle for vaccine development.

A potentially more practical approach is the RTS, S vaccine, which consists of a portion of the abundant circumsporozoite protein engineered into a hepatitis B virus like particle and adjuvanted with the GlaxoSmithKline monophosphoryl lipid A-containing AS01 adjuvant. RTS,S has been developed through a sustained public–private partnership over a period of more than 20 years. Recently, it was shown in international, multi-centre efficacy trials that this vaccine provided approximately 30–50% protection against disease in infants and young children, respectively [50,51]; protection waned considerably after 1 year [52]. This is a notable achievement and could provide a valuable tool by which to lower the burden of disease. However, if vaccine efficacy and the duration of protection could be substantially increased, the potential for impact would increase considerably.

Is it possible to achieve greater efficacy and to extend the durability of RTS,S by applying approaches similar to those proposed to generate HIV bNAbs? As noted in §3, immunological principles suggest that affinity maturation would be favoured by a progressive rationing of the antigenic target with booster immunizations, using the adjuvant formulation to maintain a robust Tfh response and to provide cytokines and direct signals to imprint memory B and plasma cell longevity. Interestingly, early in the development of RTS,S such an experiment may have been conducted serendipitously, when adverse side effects after the second immunization of a three dose regimen led to the reduction of the final dose to one-fifth of that usually employed and a longer interval between the second and third immunization. The protection observed in that human challenge study was 6 of 7, whereas protection with the standard full dose regimen in other studies has been approximately 50% [53,54]. Whether the difference in protection is due to the reduced dose, longer interval between the second and third immunization or another confounder would need to be determined in a controlled comparative study.

By contrast to the absence of vaccines for HIV and malaria more than 30 and 100 years, respectively, since the causative agents were identified, pertussis vaccines consisting of formalin-inactivated whole bacterial cells of *Bordetella pertussis* (wP) have been in use for 100 years, co-formulated with tetanus and diphtheria toxoids adsorbed on alum in DTwP vaccines since the 1940s [55]. The use of these vaccines was associated with a marked decrease in pertussis and pertussis-related mortality. However, these vaccines were too reactogenic for use in adults and older children, and even in infants and young children were associated with adverse local, systemic and neurological reactions. These reactions led to reduced uptake of vaccines in some settings, particularly in the UK, and a reciprocal increase in rates of pertussis [56]. Consequently, efforts were made to create less reactogenic vaccines, culminating in the development of acellular vaccines composed of one to five purified pertussis proteins, which were co-formulated with tetanus and diphtheria toxoids adsorbed to alum as DTaP. First licensed in Japan, several such vaccines were evaluated in comparative head-to-head immunogenicity and efficacy trials in several European countries and Senegal in the 1990s. The cumulative import of these trials was that DTaP was safe and effective, although the absolute degree of protection of the various DTaP and DTwP vaccines varied between trials as did their relative efficacy [55,57]. Because of their reduced reactogenicity, aP-containing vaccines were licensed and broadly replaced wP-containing vaccines in high-income countries. With emerging evidence that adolescents and adults were also susceptible to pertussis and transmitted the infection to infants, a booster vaccine (TdaP) for use in older children and adults and in maternal immunization efforts [58] was also developed and licensed. Due to higher costs, the switch to aP-containing vaccines was restricted to high-income countries.

Notably, the length of follow-up in these trials of aP-containing vaccines did not extend beyond 2 years. And, in the past several years, an increasing body of ecological and epidemiological data have suggested that the durability of protection induced by DTaP is shorter than that of DTwP [57,59]. In response to this information, the US Advisory Committee on Immunization Practices now recommends that all mothers, regardless of their prior immunization status, be given a TdaP booster during the third trimester to ensure that titres of protective antibodies are sufficient to transfer protection to their soon to be born infants [60]. With efforts underway to include vaccines in addition to tetanus in maternal immunization programmes in low- and middle-income countries (LMICs), the durability of protection provided by aP-containing vaccines is particularly critical due to the difficulty and costs of delivering aP-containing vaccines with each pregnancy. This concern may be partly ameliorated by evidence suggesting that the durability of protection following an aP booster is greater in individuals initially immunized with wP-containing vaccines [61], which would be the case in previously immunized mothers in LMICs; however, the magnitude of such a benefit in practice is as yet unclear.

The causes for the reduced durability of aP- compared with wP-containing vaccines are not known. Possibilities include differences in the quality (e.g. induction of Th17 and Th1 T-cell responses in addition to antibody responses by wP- but not aP-containing vaccines), magnitude and breadth of the immune responses they induce. Both the reactogenicity of wP vaccines and their ability to induce more durable and qualitatively different immune responses appear to be related at least in part to the intrinsic adjuvanticity of *B. pertussis* whole cells, which contain lipo-oliogosaccharide and other innate immune agonists not present in aP vaccines. Such differences in the immune response may also allow genetic variation in strains causing disease (e.g. loss of pertactin expression, differences between the sequence of epitopes present in the vaccine versus those in circulating strains) to more readily evade immunity induced by aP- compared with wP-containing vaccines. In any case, there is considerable impetus to develop pertussis vaccines that are less reactogenic than wP, can be safely given to pregnant women, reduce shedding and transmission (thereby providing herd immunity) and induce more durable immunity than currently licensed aP vaccines. There is some evidence in adults that immunization with a genetically detoxified pertussis toxin rather than chemically inactivated pertussis toxin, which is the form contained within existing aP vaccines, induces more potent and durable antibody responses [62]. Other approaches are under investigation. For example, a team in Argentina has created a wP-like aP vaccine consisting of outer membrane vesicles from a recombinant strain of *B. pertussis* in which the lipid A molecule is rendered less reactogenic by reducing its acylation [63]; this vaccine induces more effective and durable immunity in mice, but whether this is true in humans is not known.

A major barrier to advancing any new pertussis vaccine is the lack of immunological (or other) correlates of protection. While stringent regulatory agencies have licensed the TdaP vaccines based on their ability to boost antibody responses, they did so based on the comparability of the pertussis antigens and antibody assays to those used in that specific manufacturer's DTaP vaccine licensure trials in which protective efficacy was directly demonstrated in infants [64]. In other words, if a vaccine of different antigenic composition, different nature (e.g. live attenuated) or from a different manufacturer is to be developed, a large and perhaps impossible to perform efficacy trial would be needed.

How might this barrier be addressed? If correlates of protection that applied more broadly to aP vaccines (and ideally to other pertussis vaccines) could be defined, future vaccines could be licensed based on their immunological non-inferiority and safety without a need for a large efficacy trial. Such an approach has been used for example to license bacterial polysaccharide-conjugate vaccines and, more recently, meningococcal serogroup B protein vaccines from two different manufacturers [65,66]. But how might correlates be identified for new pertussis vaccines for which no such precedent exists without conducting a human efficacy trial? Recently, a baboon model has been developed in which pertussis can be induced either by direct inoculation or by transmission from one infected baboon to another [67]. In this model, whereas both wP and aP vaccines protect against disease, wP but not aP reduces the rate of infection, the duration of bacterial shedding in the respiratory tract and transmission to other baboons [68]. Notably, aP induces antibody responses to its constituent antigens that are greater than or equal to those induced by wP. By contrast, wP and prior infection induce antigen-specific CD4 T cells that produce IFN-γ and IL-17 but little IL-5, whereas aP induces the inverse cytokine profile. These results are consistent with those obtained in other animal models and with the more limited human data. However, whether these differences are causal and consistently associated with protection is uncertain. The application of the contemporary immunological tools described above for HIV and malaria in this model, and bridging of such studies to potential human challenge models, appear to provide a pathway to accelerate the discovery and development of more durable and effective pertussis vaccines and the correlates of protection through which they can be registered. The Innovative Medicines Initiative of the European Commission has issued a request for proposals to this end (http://www.imi.europa.eu/content/stage-1-14).

## 6. Concluding remarks

There remain great unmet needs to develop vaccines for globally burdensome infectious diseases and to allow more timely responses to emerging infectious disease threats. Vaccine development has for too long been forced to rely on empiricism due in part to a lack of commitment from other scientific disciplines to the application of the principles and tools arising from their work to rationally engineer and test new vaccines. Vaccine research and development will advance most effectively through the concerted application of these tools and principles and through collaborative alliances between vaccinology, immunology, structural, computational and systems biology, bioengineering, mathematical modelling and consortia of committed academic, industry, regulatory, governmental and funding partners.
